# Sequencing of Betacoronavirus erinacei from faeces of pet hedgehogs demonstrates a continuity of MERS-CoV like viruses in European and Eurasian hedgehog species

**DOI:** 10.1016/j.onehlt.2026.101412

**Published:** 2026-04-23

**Authors:** Gabriele Ratti, Rachael E. Tarlinton, Emanuele Lubian, Rosita Semenza, Stefania Lauzi

**Affiliations:** a Department of Veterinary Medicine and Animal Science, University of Milan, Via dell'Università 6, 26900 Lodi, Italy; b School of Veterinary Medicine and Science, University of Nottingham, Sutton Bonington Campus, Loughborough LE12 5RD, United Kingdomc. Mypetclinic, viale Ranzoni 10, 20149 Milano, Italy; c Mypetclinic, viale Ranzoni 10, 20149 Milano, Italy; d Clinica veterinaria città di Vigevano, via dei mille 22, 27029 Vigevano, Italy; e Studio veterinario dott.ssa Rosita Semenza, via Giacomo Matteotti 37, 28070 Garbagna novarese, Italy

**Keywords:** *Hedgehog coronavirus 1*, Erinaceus coronavirus, EriCoV, Coronaviruses, Exotic pet

## Abstract

Hedgehogs have been recently identified as carriers of *Betacoronavirus erinacei* (also known as Erinaceus coronavirus, EriCoV) a virus closely related to *B. cameli* responsible for human Middle East Respiratory Syndrome (MERS), raising questions about the risk of hedgehog-to-human transmission and suggesting the need for coronavirus (CoV) surveillance in hedgehogs. This study investigated the presence of CoVs in fecal samples of hedgehogs kept as pets in Italy in 2021–2022. A pan-CoV nested RT-PCR targeting the RdRp gene was used for screening and positive samples were sequenced and phylogenetically analyzed. Two (6.2%) out of 30 hedgehogs analyzed were positive for *B. erinacei* represented by 2/3 (66.7%) long eared hedgehog *(Hemiechinus auritus*) while all the 27 tested African pygmy hedgehog *(Atelerix albiventris*) were negative. Whole genome sequence obtained from one *B. erinacei*-positive sample showed closest homology (85.7%) with *B. erinacei* previously detected in *Erinaceus* sp. from Eastern Russia. Phylogeny showed that the virus of this study formed a separate clade in the cluster with other *B. erinacei* identified in Europe and European Russia and did not cluster with other *B. erinacei* identified in China in Amur hedgehog (*E. amurensis)*. No recombination events were observed. Analysis of the Spike protein revealed the presence of six out of the 11 key receptor binding residues, including two out of the three critical residues recently identified for the binding of *Erinaceus europaeus* receptor APN and *B. erinacei*. Results of this study suggest the presence of a long-eared hedgehog-specific strain of *B. erinacei*. Overall results support the circulation of coronaviruses along a phylogenetic continuum among different species of hedgehogs and geographic locations, suggesting the need for further CoV surveillance in both domestic and wild animals. There is also a need for studies on the affinity of EriCoV with the *H. auritus* APN specific receptor to confirm its involvement in the viral entry process.

## 1 Introduction

Coronaviruses (CoVs) are RNA viruses capable of causing disease in many vertebrate hosts. During the last decades a considerable number of novel viral species have been identified [1]. The family *Coronaviridae*, subfamily *Orthocoronavirinae*, contains the four genera *Alpha-*, *Beta-*, *Gamma-* and *Deltacoronavirus*. Members of the genus *Betacoronavirus* have emerged worldwide as highly pathogenic viruses in humans causing severe respiratory infections [1]. The viral species *Betacoronavirus pandemicum* (previously classified as Severe acute respiratory syndrome-related coronavirus, SARS-CoV), belonging to the subgenus *Sarbecovirus*, emerged in 2002 in China and was responsible for the severe acute respiratory syndrome (SARS) epidemic, caused by the SARS-CoV. The *Betacoronavirus cameli* (previously classified as Middle East respiratory syndrome-related coronavirus MERS-CoV), subgenus *Merbecovirus* emerged in 2012 in Saudi Arabia, causing the MERS epidemic. The recent global coronavirus disease 2019 (COVID-19) pandemic was caused by SARS-CoV-2 *(B. pandemicum*) [1], [2].

**Fig. 1:**
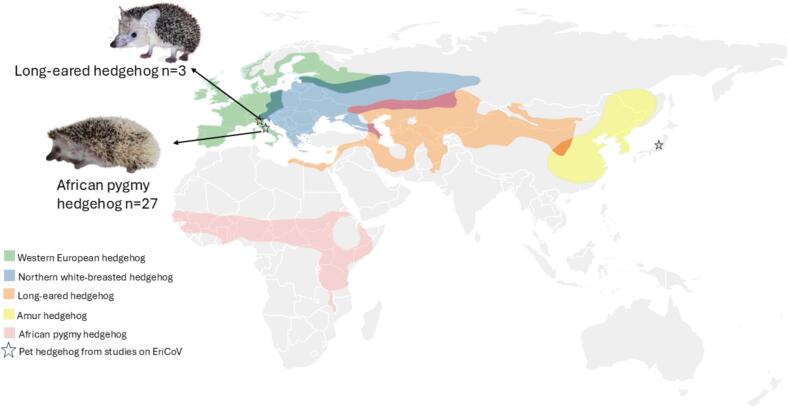
Distribution map of selected hedgehog species and hedgehogs analyzed in this study, based on the spatial data provided by the IUCN red list [17], [18], [19], [20], [22]. (For interpretation of the references to colour in this figure legend, the reader is referred to the web version of this article.)

All these three CoVs have emerged in humans through spillovers from animal hosts [3]. Following the SARS epidemic, many bat coronaviruses have been described in insectivorous bats (order *Chiroptera*). The presence of coronaviruses has also been observed in hedgehogs, animals that are phylogenetically related to the order *Chiroptera* [4], [5]. Early studies have investigated the presence of CoVs in wild hedgehogs [5]. The Erinaceus coronavirus (EriCoV), belonging to the viral species *Betacoronavirus erinacei* (previously classified as *Hedgehog coronavirus 1*) subgenus *Merbecovirus* was described for the first time in wild European hedgehogs (*Erinaceus europaeus*) from Germany, showing a close relationship with *B. cameli* and bat coronaviruses [5]. Subsequently, the presence of EriCoV has been reported in wild European hedgehogs from France, United Kingdom, Italy, Poland, Portugal and European regions of Russia [6], [7], [8], [9], [10], [11], [12]. Besides the EriCoV detected in European hedgehogs, two other phylogenetically distinct EriCoV have been reported in wild Northern white-breasted hedgehogs (*Erinaceus roumanicus*) in Hungary and in wild Amur hedgehogs (*Erinaceus amurensis*) from China, supporting the idea that there may be geographical and host species distinct lineages of hedgehog coronaviruses [13], [14], [15]. Several species of hedgehogs are widespread in Europe and other countries and have occasionally become synanthropic animals in rural areas and small towns, where they may come into contact with humans [10], [11]. Among the most common species, the African pygmy hedgehogs (*Atelerix albiventris*) are distributed in Africa, the European hedgehogs also known as the Western European hedgehogs, and the Northern white-breasted hedgehogs (*E. roumanicus)* and their hybrids are distributed across Europe and parts of Central Asia (e.g., western Siberia and Kazakhstan), the Amur hedgehogs are naturally found in East Asia, and the long-eared hedgehogs (*Hemiechinus auritus*) occur in Central and Western Asia [16], [17], [18], [19], [20], [21], [22]. These geographical ranges show partial overlap (Fig. 1), raising interest in the possible role of ecological and geographic factors in coronavirus host specificity and transmission dynamics [5], [13], [14].

Imported “exotic” species of hedgehogs from outside of Europe are frequently traded and increasingly kept as pets in Europe [23], [24], [25], [26], with the majority of hedgehog pets being African pygmy hedgehogs. To date, only one study has investigated the presence of CoVs in African pygmy hedgehogs kept as pets in Japan, reporting the absence of CoV RNA in all the tested animals [27]. Considering the potential cross-species transmission risks between EriCoV and other mammal species [10], [28] and given the close contact between human and their pets within the household environment, the need of coronavirus surveillance in hedgehog pets is warranted. Therefore, the aims of this study were i) to investigate the presence of CoVs in hedgehogs kept as pets in Italy and ii) to genetically characterize the detected CoVs using whole genome sequencing analysis.

## 2 Materials and methods

### 2.1 Animals and samples collection

This study investigated the presence of CoVs in pet hedgehogs by convenience sampling. Fecal samples were collected from privately owned hedgehogs from leftover material submitted to the Veterinary Teaching Hospital (VTH) of Lodi, University of Milan, Italy or laboratory for diagnostic purposes from 2021 to 2022. Residual fecal samples from hedgehogs collected for diagnostic purposes at the VTH with the informed consent of the owners were used for this study without any additional formal request for authorization, according to the decision of the Ethical Committee of the University of Milan (EC decision 29 October 2012, renewed with protocol no. 02–2016).

Samples were stored in sterile container at 4 °C until arrival at the laboratory (within 24 h from collection). Samples were subsequently stored at −80 °C until molecular analysis. Data regarding signalment (species, sex and age), geographic localization within Italy (Northern or Southern) clinical history (healthy or unhealthy) and management (type of housing and outdoor access) were retrieved. For the purpose of this study age was divided into two categories: juvenile (< 1 year old) and adult (≥ 1 year old) [29].

### 2.2 RNA extraction, RT-PCR protocols, sequencing and phylogeny

RNA from fecal samples was extracted using a commercial NucleoSpin viral RNA isolation kit (Macherey-Nagel, Bethlehem, PA, USA), following the manufacturer's instructions. Samples were subjected to RNA quality control targeting the vertebrate 12S rRNA gene [30].

Extracted RNA was subjected to a nested RT-PCR targeting CoV partial sequence of the RNA-dependent RNA polymerase (RdRp) gene, as previously described [31]. Briefly, the outer reaction was set up using a commercial kit (LeGene One-Step RT-PCR, Biosciences, San Diego, CA, USA) and the following primer pairs: forward: AARTTYTAYGGHGGYTGG; reverse: GARCARAATTCATGHGGDCC [32]. The inner reaction was set up using Phire Green Hot Start II PCR Master Mix (Thermo Scientific™, Segrate, Italy) and the following primer pairs: inner forward: GGTTGGGACTATCCTAAGTGTGA; inner reverse: CCATCATCAGATAGAATCATCAT [33], [34]. In each reaction, Feline Coronavirus (FCoV) RNA previously sequenced and obtained from a field sample from a cat from our laboratory was used as the positive control and RNase-free water as the blank control. The PCR was considered positive in the presence of a 440 bp amplicon in the second round of the nested RT-PCR.

PCR products were visualized under a UV transilluminator on a 1.5% agarose gel stained with ethidium bromide. The amplicons were purified using a commercial kit (NucleoSpin Gel and PCR Clean-up, Macherey-Nagel, Germany) following the manufacturer's instruction and Sanger sequenced (Microsynth Seqlab, Germany) using the inner forward and inner reverse primers used for nested RT-PCR amplification. The sequences were compared with those available in GenBank using BLAST (http://blast.ncbi.nlm.nih.gov/Blast.cgi; accessed 18 January 2026).

Partial RdRp sequences obtained from this study and selected reference sequences of other coronaviruses retrieved from NCBI dataset (accessed on 18 January 2026) were aligned and used to build the phylogenetic tree. Sequences were aligned using Clustal X; manual editing was performed with Bioedit software version 7.0 (freely available at http://www.mbio.ncsu.edu/bioedit/bioedit.html).

Phylogeny was estimated by maximum likelihood (ML) methods using MEGA v.12 [35], [36], [37]. The model used for phylogeny based on ML method was chosen according to the lowest AIC value. The robustness of the tree topology was evaluated after 1000 bootstrap replicates. For sequence comparisons, the percentage nucleotide similarity of pairwise evolutionary distances between the two CoV partial RdRp sequences of this study was calculated using MEGA v.12.

### 2.3 Whole genome sequencing and complete genome assembly

Positive samples were sent for whole genome sequencing, and mRNA sequencing was performed on one positive sample (with sufficient quality RNA) by Novogene (Novogene Company Limited, United Kingdom) with the Illumina NovaSeq 6000 platform using a library preparation and protocol for unenriched mRNA sequencing. Quality filtering, adapters, duplicates and low-quality read removal was performed using fastp v 0.23.4 [38]. Read taxonomic classification was performed using Kraken2 v2.1.2 and the viral Refseq database [39].

Reads were assembled using coronaSPAdes v 3.15.5 [40]. CheckV v 1.0.3 was used for quality assessment of contigs and genome completeness [41]. The assembled genome was annotated using Geneious Prime v2023.2 using NCBI coronavirus reference sequences of EriCoV (Taxonomy ID: 1965093).

### 2.4 Genome analysis and phylogeny

The genome sequence was compared with those available in GenBank using BLAST (http://blast.ncbi.nlm.nih.gov/Blast.cgi; accessed 18 January 2025).

The complete spike gene and complete genome sequence from CoV detected in this study, selected reference sequences of other *Merbecovirus* and all available complete nucleotide genome sequences of EriCoVs retrieved from the NCBI database (accessed on 18 January 2025) were aligned using Clustal X and manual editing was performed with Bioedit software version 7.0 (freely available at http://www.mbio.ncsu.edu/bioedit/bioedit.html) [42]. Maximum likelihood phylogenetic trees were reconstructed using IQ-TREE v 2.3.6 [43] using 1000 bootstrap approximations following implementation of UFBoot2 within IQ-TREE v 2.3.6 [44]. Phylogenetic trees were visualized in FigTree v1.4.4 (https://github.com/rambaut/figtree/).

Additionally, the nucleotide sequence of the S gene was translated into amino acids using Bioedit software version 7.0 and analyzed for the presence of the 18 key residues of the receptor binding domain (RDB) critical for binding of MERS like viruses to the human cellular receptor type II transmembrane protein, hDPP4 [45]. The amino acid sequence of the S protein RBD was also analyzed for the presence of the 11 key residues of the RDB important for binding of EriCoV to the *Erinaceus europaeus* receptor Aminopeptidase N (APN) [46].

### 2.5 Genome recombination analysis

To detect recombination events among the assembled genome, complete genome sequences were analyzed using RDP5 v5.45 using multiple different methods: RDP, GENECONV, Bootscan, Maxchi, Chimaera, SiSscan, 3Seq, and LARD [47]. Only recombination events detected by at least six methods were considered significant, as previously described [48].

### 2.6 Power calculations

The estimated number of African Pygmy hedgehogs that would need to be sampled to be reasonably sure of detecting disease at the 10.8% rates reported in wild European hedgehogs [7] was estimated using Epitools (sample size to estimate a simple proportion, precision level 0.05 and confidence interval of 0.95) with an estimate of 149 (for a large population) (https://epitools.ausvet.com.au/).

## 3 Results

### 3.1 Betacoronavirus erinacei detection

In total, fecal samples obtained from 30 domestic hedgehogs in 2021–2022 were analyzed in this study, including samples from 27 African pygmy hedgehogs and three long-eared hedgehogs (Fig. 1). Animals were all captive and housed in cages with no outdoor access. Demographic characteristics of the hedgehogs sampled are summarized in Table 1.

**Table 1:** Characteristics of hedgehogs analyzed in this study and EriCoV positivity.

Population characteristics		No. (%)	EriCoV positive no. (%)
Species	African pigmy hedgehog	27 (90)	–
	Long-eared hedgehog	3 (10)	2 (67)
Sex^a^	Male	18 (60)	1 (5)
	Female	8 (27)	1 (12)
Age^b^	<1 year	12 (40)	2 (17)
	≥1 year	13 (43)	–
Geographic localization	Northern Italy	19 (63)	2 (10)
	Southern Italy	11 (37)	–
Health status	Clinically healthy	13 (43)	2 (15)
	Unhealthy	17 (57)	–
Type of housing	Single	20 (67)	–
	Group	10 (33)	2 (20)

- negative results (0).

a Sex was unknown for four hedgehogs.

b Age was unknown for five hedgehogs.

Overall, CoVs were detected in fecal samples by nested RT-PCR in 2/30 (6.7%; 95% confidence interval: 0.8–22.0%) of the tested hedgehogs. Particularly, CoVs were detected in 2/3 (66.7%) long-eared hedgehogs from the same litter born from wild-caught individuals and housed in the same cage, whereas all African pygmy hedgehogs were negative.

**Fig. 2:**
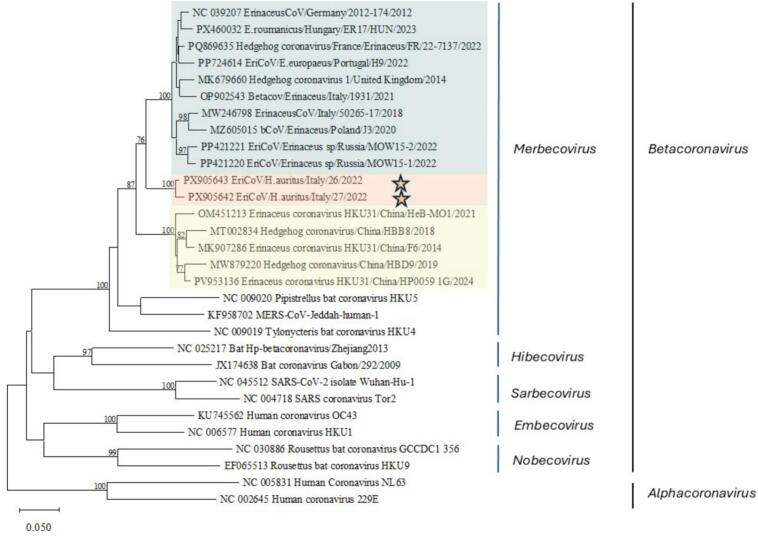
Phylogenetic tree generated with ML analysis constructed with 1000 bootstrap based on partial RdRp sequences (440 bp) obtained from CoVs belonging to the genus *Betacoronavirus* retrieved from GenBank databases and the two EriCoVs from this study. Partial RdRp sequences from Alphacoronaviruses were used as outgroup. Sequences are indicated by GenBank accession number (available at www.ncbi.nlm.nih.gov/pubmed/) and name of isolate. General Time Reversible and gamma distribution with invariant sites (G + I) model was used as parameters of nucleotide substitution, and the analytical procedure encompassed 30 nucleotide sequences with 440 positions in the final dataset. Bootstrap values >70% are shown. Sequences from this study are highlighted with an orange star and are represented by light orange colour background. Light green represents EriCoV from Western European hedgehogs and/or White-breasted hedgehogs from Europe and European Russia, and light yellow represents EriCoVs from China. (For interpretation of the references to colour in this figure legend, the reader is referred to the web version of this article.)

The two positive samples were named EriCoV*/H. auritus* 26/Italy/2022 and EriCoV*/H. auritus* 27/Italy/2022. The homology between the two CoV partial RdRp sequences from this study was 98.6%. BLAST analysis showed 87.3–91.6% and 87.5–90.7% nucleotide homology with *B. erinacei* (EriCoV) sequences deposited in GenBank for EriCoV*/H. auritus* 26/Italy/2022 and EriCoV*/H. auritus* 27/Italy/2022, respectively. The highest nucleotide similarity for both partial RdRp sequences detected in this study was observed with *B. erinacei* sequences obtained from European hedgehogs/Northern white-breasted hedgehogs from Europe and European Russia and the lowest homology was observed with EriCoV from Amur hedgehogs from China. According to phylogeny the partial RdRp sequences obtained from this study clustered within the subgenus *Merbecovirus*, clade *B. erinacei* sequences from Europe and European Russia but not from China (Fig. 2).

### 3.2 Whole genome sequencing, genome organization and recombination analysis

Of the two samples sent for RNA sequencing, only one exhibited sufficient quality to undergo sequencing, namely the EriCoV/*H. auritus* 27/Italy/2022. Kraken taxonomic classification of the sample EriCoV/*H. auritus* 27/Italy/2022 showed the presence of reads belonging to the *Coronaviridae* family. De novo assembly of the reads yielded one coronavirus contig (100% quality, 100% completeness) of 30,481 bp. BLAST analysis showed that the whole genome of the hedgehog CoV detected in this study possessed nucleotide identity ranging from 83.3% to 85.7% with other EriCoV whole genome sequences from Europe, European Russia and China, showing the highest similarity (85.7%) to EriCoVs previously found in European/Northern white-breasted hedgehogs from European Russia (GenBank accession number PP421220 and PP421221). The lowest homology of 83.3% was observed with EriCoVs from Amur hedgehogs from China (GenBank accession numbers MK907286 and OM451213).

**Fig. 3:**
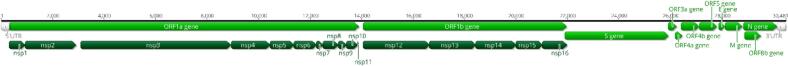
Genome organization of EriCoV detected in this study.

Genome annotation showed a typical organization of *Betacoronavirus* consisting of 5′ and 3′ untranslated regions (UTR), ORF1ab encoding for 16 non-structural peptides, genes encoding four structural proteins (spike —S—, membrane –M-, envelope –*E*- and nucleocapsid —N—) and several accessory genes (ORF3a, ORF4a, ORF4b, ORF5 and ORF8b) (Fig. 3 and supplementary table 1). BLAST analysis showed a lower nucleotide homology of the complete S gene with all the other EriCoVs compared to whole genome sequences, ranging from 79.7% to 81.7%.

Whole genome analysis did not show the presence of the additional ORF CD200 ortholog observed in some Italian strains [8].

**Fig. 4:**
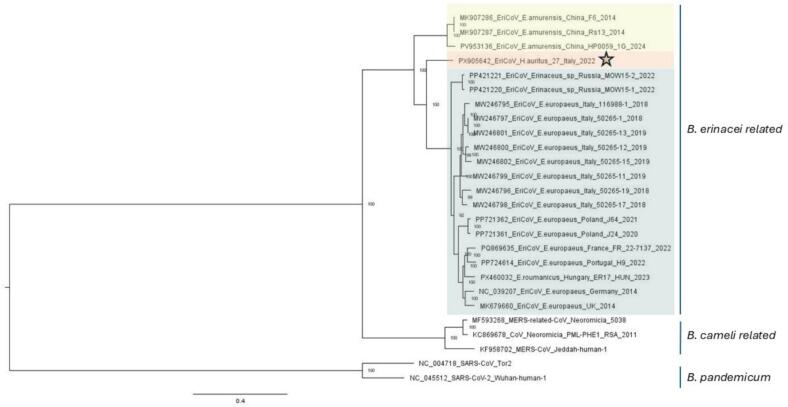
Phylogenetic tree generated with maximum likelihood analysis constructed with 1000 bootstrap based on whole genome and partial whole genome sequences from EriCoVs retrieved from GenBank databases and the EriCoV obtained in this study. *Betacoronavirus pandemicum* whole genome sequences, subgenus *Sarbecovirus* were used as outgroup. Sequences are indicated by GenBank accession number (available at www.ncbi.nlm.nih.gov/pubmed/) hedgehog species of origin, geographical origin, name of the strain and date of sample collection. The sequence from this study is highlighted with an orange star and is represented by light orange colour background. Light green represents EriCoV from Western European hedgehogs and/or Northern white-breasted hedgehogs from Europe and European Russia, and light yellow represents EriCoVs from China. (For interpretation of the references to colour in this figure legend, the reader is referred to the web version of this article.)

The phylogenetic trees based on whole genome sequences (Fig. 4) confirmed the findings of the partial RdRp sequence, showing that the sequence from this study clustered with other EriCoV sequences identified in Europe and European Russia from *Erinaceus* sp. but not with Chinese hedgehogs. More precisely, the EriCoV/*H. aritus* 27/Italy/2022 sequence from this study formed a separate clade compared to all viruses from European and European Russian hedgehogs. Similar results were observed in the phylogenetic analysis of the complete Spike gene (Supplementary Fig. 1)*.*

Recombination events were not observed in the whole genome sequence from this study.

### 3.3 Spike protein sequence analysis

Comparison of RBD of the S protein of the EriCoV/*H. auritus* 27/Italy/2022 strain and other European, European Russian and Chinese EriCoV based on the analysis of the 18 critical residues identified in previous studies for hDPP4 and MERS-CoV binding [45], [49], [50], showed that five residues (Y498, D532, D534, Y535 and V547) were found in the EriCoV/*H. auritus* 27/Italy/2022 strain, similarly to other EriCoVs analyzed (Table 2). On the other hand, six other residues out of these 18 critical residues were identical in all analyzed EriCoVs, although amino acid variability was also observed among the remaining of the 18 critical residues. Out of the 11 key residues reported for *E. europaeus* APN receptor and EriCoV from European hedgehogs*,* the EriCoV/*H. auritus* 27/Italy/2022 strain showed the presence of six residues (R521, Y527, G529, Q533, G534, T536), including two out of the three critical residues previously identified (Table 3). The EriCoV/*H. auritus* 27/Italy/2022 strain and the EriCovs from Amur hedgehogs from China showed the highest differences among the 11 residues compared to the other analyzed EriCoVs from *Erinaceus* spp. from Europe and European-Russia.

**Table 2:** Sequence alignment showing variations in the 18 crucial key amino acid binding residues of the Spike protein. The critical residues themselves and residues responsible for critical bond formation previously identified for hDPP4 and MERS-CoV binding are labelled with * and **, respectively. White boxes refer to residues observed in EriCoV sequences identical to the MERS-CoV reference sequence. Conserved amino acid residues observed in all EriCoVs sequences differing from the reference sequence are highlighted in bold. All sequences are identified by GenBank accession numbers.

			**	*	*	*	**		**		*	*		**		*	*	*
MERS-CoV (NC_019843)	D 455	P 463	Y 499	N 501	K 502	L 506	D 510	R 511	E 513	W 535	E 536	D 537	G 538	D 539	Y 540	R 542	W 553	V 555
EriCoV/ *H. auritus* 27/Italy/2022 (PX905642, this study)	**T 451**	A 459	Y 498	**S 500**	**R 501**	T 505	**K 508**	P 509	**–**	N 530	D 531	D 532	V 533	D 534	Y 535	**G 537**	Y 545	V 547
EriCoV Italy (MW246795, MW246797, MW246800) MW246801)	**T 443**	A 451	Y 490	**S 492**	**R 493**	T 497	**-K 500**	P 501	**–**	P 522	S 523	D 524	A 525	D 526	Y 527	**G 529**	Y 537	L 539
EriCoV Italy (MW246796)	**T 444**	A 452	Y 491	**S 493**	**R 494**	T 498	**K 501**	P 502	**–**	P 523	S 524	D 525	A 526	D 527	Y 528	**G 530**	Y 538	V 540
EriCoV Italy (MW246798, MW246799)	**T 444**	A 452	Y 491	**S 493**	**R 494**	T 498	**K 500-**	P 501	**–**	Q 523	N 524	D 525	A 526	D 527	Y 528	**G 530**	Y 538	I 540
EriCoVs Italy (MW246802)	**T 444**	A 452	Y 491	**S 493**	**R 494**	I 498	**K 501-**	P 502	**–**	K 523	K 524	D 525	V 526	D 527	Y 528	**G 530**	Y 538	V 540
EriCoV Portugal (PP724614)	**T 445**	A 453	Y 492	**S 494**	**R 495**	T 499	**K 502**	S 503		P 524	S 525	D 526	A 527	D 528	Y 529	**G 531**	Y 539	L 541
EriCoV Germany (NC_039207)	**T 443**	A 451	Y 490	**S 492**	**R 493**	V 497	**K 500**	P 501	**–**	P 522	S 523	D 524	A 525	D 526	Y 527	**G 529**	Y 537	L 539
EriCov France (PQ869635)	**T 440**	T 448	Y 487	**S 489**	**R 490**	T 494	**K 497**	P 498	**–**	P 519	S 520	D 521	A 522	D 523	Y 524	**G 526**	Y 534	L 536
EriCoV UK (MK679660)	**T 442**	A 450	Y 489	**S 491**	**R 492**	T 496	**K 499**	P 500	**–**	P 521	S 522	D 523	A 524	D 525	Y 526	**G 528**	Y 536	L 538
EriCoVs Poland (PP721361, PP721362)	**T 443**	A 452	Y 490	**S 492**	**R 493**	I 497	**K 500**	P 501	**–**	P 522	S 523	D 524	A 525	D 526	Y 527	**G 529**	Y 537	L 539
EriCoV Hungary (PX460032)	**T 443**	A 451	Y 490	**S 492**	**R 493**	T 497	**K 500**	P 501	**–**	P 522	S 523	D 524	A 525	D 526	Y 527	**G 529**	Y 537	L 539
EriCoV European Russia (PP421220, PP421221)	**T 443**	A 452	Y 491	**S 493**	**R 494**	T 498	**K 501**	P 502	**–**	P 523	S 524	D 525	A 526	D 527	Y 528	**G 530**	Y 538	L 540
EriCoVs China (MK907286, MK907287)	**T 443**	A 452	Y 491	**S 493**	**R 494**	T 498	**K 501-**	P 502	**–**	S 523	N 524	D 525	V 526	D 527	Y 528	**G 530**	F 538	I 540
EriCoV China (PV953136)	**T 444**	A 452	Y 491	**S 493**	**R 494**	**T 498**	**K 501**	P 502	**–**	P 523	**N 524**	D 525	**V 526**	D 527	Y 528	**G 530**	F 538	V 540

**Table 3:** Sequence alignment showing variations in the 11 key amino acid binding residues of the Spike protein for APN hedgehog. The residues responsible for critical bond formation previously identified for APN hedgehog and EriCoV-12-19 (MW246800) binding are labelled with *. White boxes refer to amino acid residues that are identical to the ones observed in EriCoV-12-19 RBD sequence. Conserved residues observed in all analyzed sequences are highlighted in bold. All sequences are identified by GenBank accession numbers.

Zone of the RBD as described by Jin et al., 2026	1	1	1	2	3	3	3	3	3	3	2
	*			*							*
ErinCoV-12-19 RBD (MW246800)	R 521	P 522	A 525	**Y 527**	**G 529**	Y 530	S 531	Q 533	**G 534**	T 536	Y 538
EriCoV/ *H. auritus* 27/Italy/2022 (PX905642, this study)	R 529	N 530	V 533	**Y 535**	**G 537**	T 538	Q 539	Q 541	**G 542**	T 544	F 546
EriCoVs Italy (MW246795, MW246797, MW246801)	R 521	P 522	A 525	**Y 527**	**G 529**	Y 530	S 531	Q 533	**G 534**	T 536	Y 538
EriCoV Italy (MW246796)	L 522	P 523	A 526	**Y 528**	**G 530**	Y 531	S 532	Q 534	**G 535**	T 537	Y 539
EriCoVs Italy (MW246798, MW246799)	M 522	Q 523	A 526	**Y 528**	**G 530**	Y 531	S 532	Q 534	**G 535**	T 537	Y 539
EriCoVs Italy (MW246802)	M 522	Q 523	V 526	**Y 528**	**G 530**	Y 531	S 532	Q 534	**G 535**	I 537	Y 539
EriCoV Portugal (PP724614)	R 523	P 524	A 527	**Y 529**	**G 531**	Y 532	S 533	Q 535	**G 536**	T 538	Y 540
EriCoV Germany (NC_039207)	R 521	P 522	A 525	**Y 527**	**G 529**	Y 530	S 531	Q 533	**G 534**	N 536	Y 538
EriCov France (PQ869635)	R 518	P 519	A 522	**Y 524**	**G 526**	Y 527	S 528	Q 530	**G 531**	T 533	Y 535
EriCoV UK (MK679660)	W 520	P 521	A 524	**Y 526**	**G 528**	Y 529	S 530	Q 532	**G 533**	T 535	Y 537
EriCoVs Poland (PP721361)	W 521	P 522	A 525	**Y 527**	**G 529**	Y 530	S 531	Q 533	**G 534**	T 536	Y 538
EriCoVs Poland (PP721362)	R 521	P 522	A 525	**Y 527**	**G 529**	Y 530	S 531	Q 533	**G 534**	T 536	Y 538
EriCoV Hungary (PX460032)	R 521	P 522	A 525	**Y 527**	**G 529**	Y 530	S 531	Q 533	**G 535**	T 536	Y 538
EriCoVs European Russia (PP421220, PP421221)	R 522	P 523	A 526	**Y 528**	**G 530**	Y 531	T 532	Q 534	**G 535**	T 537	Y 539
EriCoVs China (MK907286, MK907287)	L 522	S 523	V 526	**Y 528**	**G 520**	Y 531	N 532	N 534	**G 535**	I 537	Y 539
EriCoV China (PV953136)	L 522	P 523	V 526	**Y 528**	**G 530**	Y 531	N 532	N 534	**G 535**	N 537	Y 539

### 3.4 Whole genome and nucleotide sequence accession numbers

Raw sequencing data have been deposited in the NCBI SRA database under BioProject accession number PRJNA1405345 and the whole genome assembly of EriCoV/*H. auritus* 27/Italy/2022 obtained in this study was uploaded to the GenBank database under accession number PX905642. The partial sequence of the RdRp gene obtained from the other positive sample not subjected to whole genome sequencing (EriCoV*/H. auritus* 26/Italy/2022) was deposited in GenBank under accession number PX905643.

## 4 Discussion

Hedgehogs have been suggested as potential natural reservoirs of coronaviruses in Eurasia [8], [9], [13]. Hence, this study investigated the presence of coronavirus in fecal samples collected in 2021–2022 from hedgehogs kept as pets in Italy, namely African pygmy hedgehog (*A. albiventris*) and long-eared hedgehog (*H. auritus*).

Detection of *B. erinacei* in hedgehogs kept as pets with an overall 6.7% EriCoV positivity observed in this study is similar to previous reports in wild hedgehogs from China and the UK [7], [14] but lower compared to other reports showing higher prevalence ranging from 58.3% to 68.5% in wild hedgehogs in Italy, Germany and France [5], [6], [10], [11], [28], [51]. However, the overall positivity detected in our study may have been influenced by the convenience sampling of this study, and it does not allow us to infer EriCoV prevalence in the population of pet hedgehogs in Italy.

Regarding positivity in hedgehog species, the results of our study showed the first identification of *B. erinacei* in long-eared hedgehogs. This result is in accordance with previous studies showing different species of wild hedgehogs as natural reservoirs of coronavirus (mainly European hedgehogs, Northern white-breasted hedgehogs and Amur hedgehogs) across Eurasia [5], [6], [7], [8], [9], [10], [11], [12], [13], [14], [15], [28], [51]. Positivity in long-eared hedgehogs may be explained by their distribution in Asia overlapping in some areas the distribution of Northern white-breasted hedgehogs and Amur hedgehogs previously found to be EriCoV-positive [11], [13], [15], [21] along with the fact that they have been kept as pets only recently [52].

On the other hand, decades of captive breeding as well as the individual housing requirement for African pygmy hedgehog [53], which may reduce the risk of CoV introduction and transmission within captive animals, may account for the absence of CoV in pet African pygmy hedgehog fecal samples observed in this study. The negative results in African pygmy hedgehogs kept as pets is in line with a previous report that did not detect CoVs using oral swabs in captive hedgehogs of this species from Japan [27]. However, to be sure of this finding, sampling a larger number of animals (estimated to be at least 149 based on an estimated prevalence of 10.8%) would be required. Further studies with a higher number of animals and adequate sampling strategies are needed to confirm the results from this study in pet hedgehogs as well as to assess the potential presence of CoVs in wild African pygmy hedgehog and long-eared hedgehog populations.

With regards to the pathogenicity of hedgehog coronaviruses, there are some studies where EriCoVs have been identified in hedgehogs with clinical signs of disease. However, the presence of EriCoV in two clinically healthy pet hedgehogs in this study is in line with the absence of correlation between EriCoV positivity and animal health conditions previously reported [28]. This suggests that betacoronaviruses are more likely to be commensal than pathogenic in hedgehogs [14], [51], [54]. The finding in our study of EriCoV in two juvenile animals aligns with previous observations showing five times higher odds of being EriCoV positive in juvenile hedgehogs [28].

The partial RdRp sequence and the whole genome sequence exhibiting features similar to other European/European Russian EriCoVs was expected and the absence of an additional ORF CD200 ortholog observed in some Italian strains [8] was not surprising and confirms previous reports not detecting its presence in other *B. erinacei* whole genome sequences [28].

The viruses identified here in long-eared hedgehogs sit phylogenetically between isolates from Asian and European/European Russia hedgehogs. They are more closely related to isolates from European Russian animals than previous Italian isolates. However, the viruses in this study were isolated from recently domesticated animals (born in captivity from wild caught parents) from a species with a host range in central Asia that overlaps with the species sampled in the European Russian study [11]. This indicates that hedgehog coronaviruses likely display a continuum across Eurasia as also demonstrated in a recent study of wild Hungarian Northern white-breasted hedgehogs [15]. The finding in our study of a separate clade within the EriCoVs in long-eared hedgehogs naturally distributed in Asia and that have not been analyzed in previous studies also suggests the presence of species-specific strains of EriCoVs, in line with previous reports showing that in general, each coronavirus has a narrowly restricted host range and coronaviruses are species-specific [55]. Sampling of further wild animals and species may likely demonstrate more viruses of this group in hedgehogs.

Host susceptibility to CoVs is determined by the interaction between the receptor-binding domain (RBD) of the S protein of coronaviruses and host cell receptor proteins [56]. The presence of several conserved amino acids of the RBD in the EriCoV from this study and all European, European Russian and Chinese EriCoVs confirms previous reports suggesting an affinity for the same receptor in hedgehog body tissues [28]. Previously it was speculated that the EriCoV receptor in host cells may be the same as for MERS-CoV, namely DPP4 [10]. However, a recent virus pseudotype and cell entry study has demonstrated that the receptor for European strains of EriCoV in hedgehogs is Aminopeptidase N (APN), a receptor used by a range of mammalian alphacoronaviruses including canine and feline alphacoronaviruses and human coronavirus HCoV-229E [46]. This is in line with many studies demonstrating low affinity of EriCoVs for hedgehog or human DDP4 [5], [7], [8], [10], [13], [28]. Moreover, recent structural evidence suggests that species-specific differences in APN may represent a significant barrier to cross-species transmission of EriCoVs. In particular, key amino acid residues required for EriCoV spike binding differ between hedgehogs and humans, resulting in steric incompatibilities that likely impair viral attachment. Additionally, differences in glycosylation patterns of human APN may introduce further steric impediment, reducing the likelihood of efficient binding [46]. Overall, these findings suggest that despite the phylogenetic relationship between EriCoV and MERS-related viruses, differences in receptor usage are likely to limit its capacity to infect humans, highlighting the importance of studying molecular receptor interactions when evaluating zoonotic risk.

The presence in the sequence from this study of key residues of the RBD similar to the ones reported for hedgehogs from Europe and European Russia suggests that hedgehog APN may be an interesting candidate for further studies. The differences in the key residues of the RBD among EriCoVs from the long-eared hedgehog of this study, Amur hedgehogs and *Erinaceus* spp. suggest that further studies should also compare RBD binding affinity with APN from long-eared and Amur hedgehogs. It is also possible that hedgehog viruses might use alternate receptors in other species with Cruz et al. 2024's protein docking studies of EriCoV RBD indicating that European EriCov shows higher binding affinity for fox and cat DPP4 and human ACE2 than the hedgehog variants of these proteins [10]. Also these receptor binding affinities need to be further explored.

Overall, the identification of *B. erinaceus* in hedgehogs kept as pets in Italy is of concern, given the close contact between human and exotic pets within the household environment and the potential cross-species transmission risks between EriCoV and other mammal species [10], [28]. Hedgehogs are among exotic animals that have become popular as pets in Europe [26], with the international trade of wild animals as pets becoming a multi-billion-dollar industry, with the EU being one of the largest markets, with an increasing demand for exotic pets [23], [24], [25]. Hedgehogs may carry several potentially zoonotic pathogens [11], [57], [58], [59] and the risks of their transmission to humans supports the debate on the role of wildlife trade as a potential source of emerging zoonotic diseases [60], further highlighting the need of continuous surveillance studies not only on CoVs in hedgehogs kept as pets.

## 5 Conclusion

In conclusion, the present study added information on coronaviruses in hedgehogs, showing circulation of EriCoV with genetic features suggesting the presence of species-specific strains of EriCoVs in long eared hedgehogs kept as pets in Italy, highlighting the need of further coronavirus surveillance in both domestic and wild animals also focused on the identification of EriCoV entry receptor in order to define the risk of hedgehog-to-human transmission of coronaviruses.

## CRediT authorship contribution statement

**Gabriele Ratti:** Writing – original draft, Methodology, Formal analysis, Data curation. **Rachael E. Tarlinton:** Writing – review & editing, Supervision, Investigation, Funding acquisition. **Emanuele Lubian:** Writing – review & editing, Methodology. **Rosita Semenza:** Writing – review & editing, Methodology. **Stefania Lauzi:** Writing – review & editing, Supervision, Project administration, Methodology, Conceptualization.

## Ethical approval

Samples were collected according to the diagnostic procedures and according to the Ethical Committee decision of the University of Milan, residual aliquots of samples or tissues collected for diagnostic purposes at the VTH under informed consent of the owners can be used for research purposes without any additional formal request of authorization (EC decision 29 Oct 2012, renewed with the protocol no. 02–2016).

## Declaration of competing interest

The authors declare that they have no conflicts of interest associated with this study. All authors reviewed, revised and approved the final manuscript and have contributed significantly to the work.

## Data Availability

The partial RdRp sequence of *H. auritus* 26 and the whole genome sequence of *H. auritus* 27 have been deposited under accession number PX905643 and PX905642, respectively. Raw sequencing data of *H. auritus* 27 have been deposited in the NCBI SRA database under BioProject accession number PRJNA1405345. The data that support the findings of this study are available from the corresponding author upon reasonable request.

## References

[1] Woo P.C.Y., de Groot R.J., Haagmans B., Lau S.K.P., Neuman B.W., Perlman S., Sola I., van der Hoek L., Wong A.C.P., Yeh S.H. (2023). ICTV virus taxonomy profile: Coronaviridae 2023. J. Gen. Virol..

[2] International Taxonomy of Viruses (ICTV) (2024). https://ictv.global/taxonomy.

[3] Ruiz-Aravena M., McKee C., Gamble A., Lunn T., Morris A., Snedden C.E., Yinda C.K., Port J.R., Buchholz D.W., Yeo Y.Y., Faust C., Jax E., Dee L., Jones D.N., Kessler M.K., Falvo C., Crowley D., Bharti N., Brook C.E., Aguilar H.C., Peel A.J., Restif O., Schountz T., Parrish C.R., Gurley E.S., Lloyd-Smith J.O., Hudson P.J., Munster V.J., Plowright R.K. (2022). Ecology, evolution and spillover of coronaviruses from bats. Nat. Rev. Microbiol..

[4] Bininda-Emonds O.R., Cardillo M., Jones K.E., MacPhee R.D., Beck R.M., Grenyer R., Price S.A., Vos R.A., Gittleman J.L., Purvis A. (2007). The delayed rise of present-day mammals. Nature.

[5] Corman V.M., Kallies R., Philipps H., Göpner G., Müller M.A., Eckerle I., Brünink S., Drosten C., Drexler J.F. (2014). Characterization of a novel betacoronavirus related to middle east respiratory syndrome coronavirus in european hedgehogs. J. Virol..

[6] Monchatre-Leroy E., Boué F., Boucher J.M., Renault C., Moutou F., Ar Gouilh M., Umhang G. (2017). Identification of alpha and beta coronavirus in wildlife species in France: bats, rodents, rabbits, and hedgehogs. Viruses.

[7] Saldanha I.F., Lawson B., Goharriz H., Rodriguez-Ramos Fernandez J., John S.K., Fooks A.R., Cunningham A.A., Johnson N., Horton D.L. (2019). Extension of the known distribution of a novel clade c betacoronavirus in a wildlife host. Epidemiol. Infect..

[8] De Sabato L., Di Bartolo I., De Marco M.A., Moreno A., Lelli D., Cotti C., Delogu M., Vaccari G. (2020). Can coronaviruses steal genes from the host as evidenced in western european hedgehogs by EriCoV genetic characterization?. Viruses.

[9] Pomorska-Mól M., Ruszkowski J.J., Gogulski M., Domanska-Blicharz K. (2022). First detection of hedgehog coronavirus 1 in Poland. Sci. Rep..

[10] Cruz A.V.S., Santos-Silva S., Queirós-Reis L., Rodrigues C., Soeiro V., Tarlinton R.E., Mesquita J.R. (2024). Genomic characterization and cross-species transmission potential of hedgehog coronavirus. One Health.

[11] Lukina-Gronskaya A.V., Chudinov I.K., Korneenko E.V., Mashkova S.D., Semashko T.A., Sinkova M.A., Penkin L.N., Litvinova E.M., Feoktistova N.Y., Speranskaya A.S. (2024). Novel coronaviruses and mammarenaviruses of hedgehogs from Russia including the comparison of viral communities of hibernating and active specimens. Front. Vet. Sci..

[12] Beissat K., Picard-Meyer E., Arné P., Chanteclair F., Le Barzic C., Hivert L., Le Loc’h G., Puech M.-P., Bastien F., Schereffer J.-L., Wasniewski M. (2025). Coding-complete genome sequences of hedgehog coronavirus isolated from *Erinaceus europaeus* in France. Microbiol. Resour. Announc..

[13] Lau S.K.P., Luk H.K.H., Wong A.C.P., Fan R.Y.Y., Lam C.S.F., Li K.S.M., Ahmed S.S., Chow F.W.N., Cai J.P., Zhu X., Chan J.F.W., Lau T.C.K., Cao K., Li M., Woo P.C.Y., Yuen K.Y. (2019). Identification of a novel betacoronavirus (merbecovirus) in Amur hedgehogs from China. Viruses.

[14] Li D., Gong X.Q., Xiao X., Han H.J., Yu H., Li Z.M., Yan L.N., Gu X.L., Duan S.H., Yu X. (2021). MERS-related CoVs in hedgehogs from Hubei province, China. One Health.

[15] Reuter G., Cora C.E., Takáts K., Boros Á., Mátics R., Balázs B., Pankovics P. (2026). Detection and molecular analysis of betacoronaviruses (family coronaviridae) in hedgehogs (erinaceus roumanicus) in Hungary. Arch. Virol..

[16] Morris P.A. (1988). A study of home range and movements in the hedgehog (erinaceus europaeus). J. Zool..

[17] IUCN (International Union for Conservation of Nature) (2008). The IUCN Red List of Threatened Species.

[18] IUCN (International Union for Conservation of Nature) (2008). The IUCN Red List of Threatened Species 2025(2).

[19] IUCN (International Union for Conservation of Nature) (2008). The IUCN Red List of Threatened Species 2025(2).

[20] IUCN (International Union for Conservation of Nature) (2008). The IUCN Red List of Threatened Species 2025(2).

[21] He K., Chen J.-H., Gould G.C., Yamaguchi N., Ai H.-S., Wang Y.-X., Wang H., Zhang Y., Jiang J. (2012). An estimation of erinaceidae phylogeny: a combined analysis approach. PLoS One.

[22] IUCN (International Union for Conservation of Nature) SSC Small Mammal Specialist Group (2023). The IUCN Red List of Threatened Species 2025(2).

[23] Check E. (2004). Health concerns prompt US review of exotic-pet trade. Nature.

[24] Engler M., Parry-Jones R. (2007).

[25] Endcap (2012). Wild Pets EU Report. https://endcap.eu/wp-content/uploads/2013/02/Report-Wild-Pets-in-the-European-Union.pdf.

[26] (2020). Eurogroup for Animals. https://www.eurogroupforanimals.org/files/eurogroupforanimals/2022-03/Eurogroup%20for%20Animals_Exotic%20pets%20reoprt_v5%20.

[27] Koizumi I., Tsukada H., Hayasaka D., Shimoda H. (2022). Comprehensive surveillance of virus infection among captive african pygmy hedgehogs in Japan. Viruses.

[28] Domanska-Blicharz K., Lisowska A., Opolska J., Ruszkowski J.J., Gogulski M., Pomorska-Mól M. (2024). Whole genome characteristics of hedgehog coronaviruses from Poland and analysis of the evolution of the spike protein for its interspecies transmission potential. BMC Vet. Res..

[29] Haigh A., Kelly M., Butler F., O’Riordan R.M. (2014). Non invasive methods of separating hedgehog (erinaceus europaeus) age classes and an investigation into the age structure of road kill. Acta Theriol..

[30] Kitano T., Umetsu K., Tian W., Osawa M. (2007). Two universal primer sets for species identification among vertebrates. Int. J. Legal Med..

[31] Drzewnioková P., Festa F., Panzarin V., Lelli D., Moreno A., Zecchin B., De Benedictis P., Leopardi S. (2021). Best molecular tools to investigate coronavirus diversity in mammals: a comparison. Viruses.

[32] Hu H., Jung K., Wang Q., Saif L.J., Vlasova A.N. (2018). Development of a one-step RT-PCR assay for detection of pancoronaviruses (α-, β-, γ-, and δ-coronaviruses) using newly designed degenerate primers for porcine and avian fecal samples. J. Virol. Methods.

[33] Poon L.L.M., Chu D.K.W., Chan K.H., Wong O.K., Ellis T.M., Leung Y.H.C., Lau S.K., Woo P.C.Y., Suen K.Y., Yuen K.Y., Guan Y., Peiris J.S.M. (2005). Identification of a novel coronavirus in bats. J. Virol..

[34] Chu D.K.W., Poon L.L.M., Chan K.H., Chen H., Guan Y., Yuen K.Y., Peiris J.S.M. (2006). Coronaviruses in bent-winged bats (miniopterus spp.). J. Gen. Virol..

[35] Kimura M. (1980). A simple method for estimating evolutionary rates of base substitutions through comparative studies of nucleotide sequences. J. Mol. Evol..

[36] Felsenstein J. (1981). Evolutionary trees from DNA sequences: a maximum likelihood approach. J. Mol. Evol..

[37] Kumar S., Stecher G., Suleski M., Sanderford M., Sharma S., Tamura K. (2024). Molecular evolutionary genetics analysis version 12 for adaptive and green computing. Mol. Biol. Evol..

[38] Chen S., Zhou Y., Chen Y., Gu J. (2018). Fastp: an ultra-fast all-in-one FASTQ preprocessor. Bioinformatics.

[39] Wood D.E., Lu J., Langmead B. (2019). Improved metagenomic analysis with kraken 2. Genome Biol..

[40] Meleshko D., Hajirasouliha I., Korobeynikov A. (2021). CoronaSPAdes: from biosynthetic gene clusters to RNA viral assemblies. Bioinformatics.

[41] Nayfach S., Camargo A.P., Schulz F., Eloe-Fadrosh E., Roux S., Kyrpides N.C. (2021). CheckV assesses the quality and completeness of metagenome-assembled viral genomes. Nat. Biotechnol..

[42] Katoh K., Standley D.M. (2013). MAFFT multiple sequence alignment software version 7: improvements in performance and usability. Mol. Biol. Evol..

[43] Trifinopoulos J., Nguyen L.T., von Haeseler A., Minh B.Q. (2016). W-IQ-TREE: a fast online phylogenetic tool for maximum likelihood analysis. Nucleic Acids Res..

[44] Hoang D.T., Chernomor O., von Haeseler A., Minh B.Q., Vinh L.S. (2018). UFBoot2: improving the ultrafast bootstrap approximation. Mol. Biol. Evol..

[45] Lu G., Wang Q., Gao G.F. (2015). Bat-to-human: spike features determining “host jump” of coronaviruses SARS-CoV, MERS-CoV, and beyond. Trends Microbiol..

[46] Jin M., Jefferson V.A., Zhao Z., Catanzaro N.J., De Sabato L., Keller E.L., Menasche B.L., Hoffman C., Castelli A., Di Bartolo I., Vaccari G., Starr T.N., Seifert S.N., Castellanos A.A., Han B.A., Serebryannyy L., Spaulding A.B., Douek D.C., Moreno A., Baric R.S., Wilen C.B., Rini J.M., Letko M. (2026). Aminopeptidase N is a receptor for hedgehog *merbecoviruses*. bioRxiv.

[47] Martin D.P., Varsani A., Roumagnac P., Botha G., Maslamoney S., Schwab T., Kelz Z., Kumar V., Murrell B. (2020). RDP5: A computer program for analyzing recombination in, and removing signals of recombination from, nucleotide sequence datasets. Virus Evol..

[48] Apaa T., Withers A.J., Staley C., Blanchard A., Bennett M., Bremner-Harrison S., Chadwick E.A., Hailer F., Harrison S.W.R., Loose M., Mathews F., Tarlinton R. (2023). Sarbecoviruses of british horseshoe bats; sequence variation and epidemiology. J. Gen. Virol..

[49] Lu G., Hu Y., Wang Q., Qi J., Gao F., Li Y., Zhang Y., Zhang W., Yuan Y., Bao J., Zhang B., Shi Y., Yan J., Gao G.F. (2013). Molecular basis of binding between novel human coronavirus MERS-CoV and its receptor CD26. Nature.

[50] Wang N., Shi X., Jiang L., Zhang S., Wang D., Tong P., Guo D., Fu L., Cui Y., Liu X., Arledge K.C., Chen Y.H., Zhang L., Wang X. (2013). Structure of MERS-CoV spike receptor-binding domain complexed with human receptor DPP4. Cell Res..

[51] Delogu M., Cotti C., Lelli D., Sozzi E., Trogu T., Lavazza A., Garuti G., Castrucci M.R., Vaccari G., De Marco M.A., Moreno A. (2020). Eco-virological preliminary study of potentially emerging pathogens in hedgehogs (erinaceus europaeus) recovered at a wildlife treatment and rehabilitation center in northern Italy. Animals.

[52] Moshaverinia A., Borji H., Kameli M., Ghabdian S., Ghanei R. (2016). A survey on parasites of long-eared hedgehog (hemiechinus auritus) in northeast of Iran. J. Parasit. Dis..

[53] Doss G.A., Carpenter J.W., Carpenter J.W., Baer C.V. (2020). Ferrets, Rabbits, and Rodents: Clinical Medicine and Surgery.

[54] De Sabato L., Ianiro G., Manzia F., Monini M., Chiappini B., Di Bartolo I., Vaccari G. (2023). Erinaceus coronavirus persistence in hedgehogs (erinaceus europaeus) in a non-invasive, in vivo, experimental setting. Front. Vet. Sci..

[55] Shehata A.A., Attia Y.A., Rahman M.T., Basiouni S., El-Seedi H.R., Azhar E.I., Khafaga A.F., Hafez H.M. (2022). Diversity of coronaviruses with particular attention to the interspecies transmission of SARS-CoV-2. Animals.

[56] Li F. (2015). Receptor recognition mechanisms of coronaviruses: a decade of structural studies. J. Virol..

[57] Smith A.J. (1999). Husbandry and nutrition of hedgehogs. Vet. Clin. North Am. Exot. Anim. Pract..

[58] Keeble E., Koterwas B. (2020). Selected emerging diseases of pet hedgehogs. Vet. Clin. North Am. Exot. Anim. Pract..

[59] Ruszkowski J.J., Hetman M., Turlewicz-Podbielska H., Pomorska-Mól M. (2021). Hedgehogs as a potential source of zoonotic pathogens—a review and an update of knowledge. Animals.

[60] Shivaprakash K.N., Sen S., Paul S., Kiesecker J.M., Bawa K.S. (2021). Mammals, wildlife trade, and the next global pandemic. Curr. Biol..

