# Supplementary material for: Sequencing of Betacoronavirus erinacei from faeces of pet hedgehogs demonstrates a continuity of MERS-CoV like viruses in European and Eurasian hedgehog species
Source: One Health. 2026 Apr 23;22:101412. doi: 10.1016/j.onehlt.2026.101412 (PMC13157107; doi:10.1016/j.onehlt.2026.101412)
Supplement: Supplementary file 1 — Supplementary material [file mmc1.docx]

**Supplementary Figure 1**. Phylogenetic tree generated with maximum likelihood analysis constructed with 1000 bootstrap based on complete S gene sequences from EriCoVs retrieved from GenBank databases and the EriCoV obtained in this study. *Betacoronavirus pandemicum*, subgenus *Sarbecovirus* were used as outgroup. Sequences are indicated by GenBank accession number (available at www.ncbi.nlm.nih.gov/pubmed/) hedgehog species of origin, geographical origin, name of the strain and date of sample collection.. The sequence from this study is highlightedwith an orange star and is represented by light orange color background. Light green represents EriCoV from Western European hedgehogs and/or Northern white-breasted hedgehogs from Europe and European Russia, and light yellow represents EriCoVs from China.


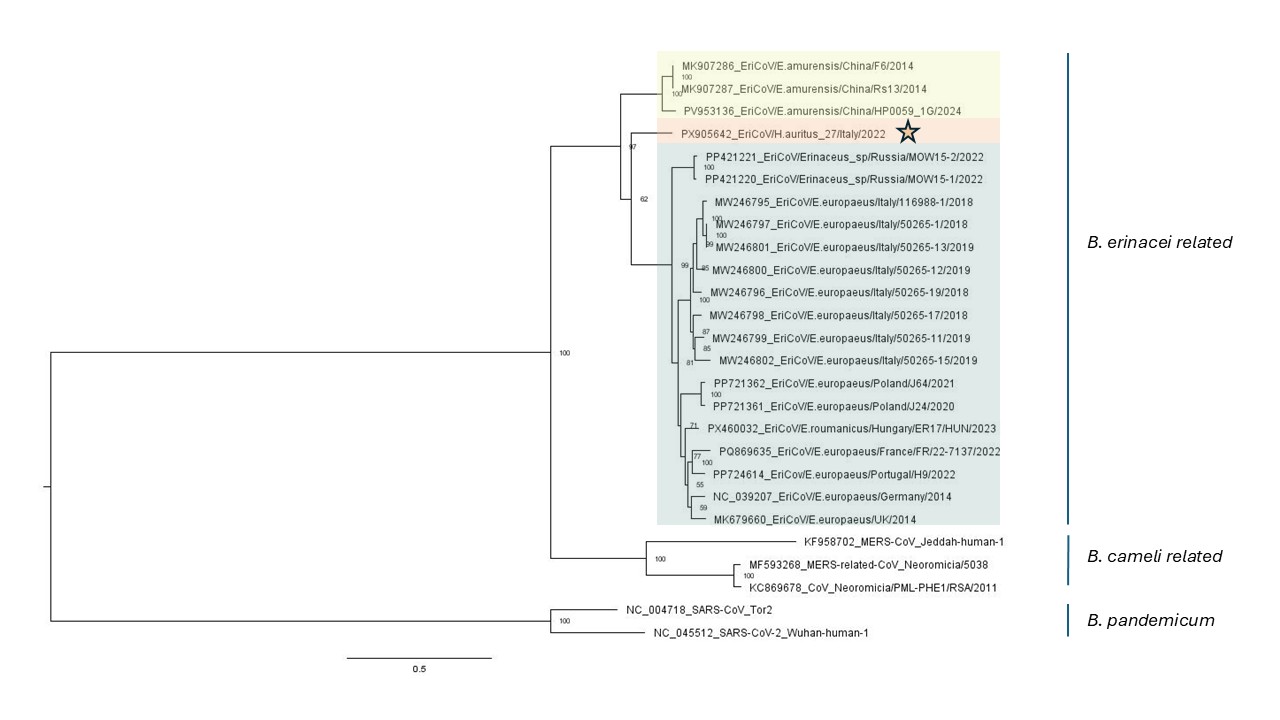


**Supplementary Table 1**. Location and length of genes of the EriCoVs detected in this study.

| Gene | Location in the genome | Length |
| --- | --- | --- |
| 5'UTR | 1-322 | 322 |
| ORF1ab | 323-21972 | 21650 |
| ORF1a gene | 323-13882 | 13563 |
| ORF1b gene | 14074-21948 | 7875 |
| nsp1 | 323-922 | 600 |
| nsp2 | 923-2899 | 1977 |
| nsp3 | 3089-8929 | 5841 |
| nsp4 | 8936-10450 | 1515 |
| nsp5 | 10451-11368 | 918 |
| nsp6 | 11369-12244 | 876 |
| nsp7 | 12245-12493 | 249 |
| nsp8 | 12494-13090 | 597 |
| nsp9 | 13091-13420 | 330 |
| nsp10 | 13421-13840 | 420 |
| nsp11 | 13841-13882 | 42 |
| nsp12 | 14074-16641 | 2568 |
| nsp13 | 16642-18435 | 1794 |
| nsp14 | 18436-20007 | 1572 |
| nsp15 | 20008-21036 | 1029 |
| nsp16 | 21037-21948 | 912 |
| S | 21890-25903 | 4014 |
| ORF3a | 25920-26237 | 318 |
| ORF4a | 26191-26439 | 249 |
| ORF4b | 26428-27106 | 708 |
| ORF5 | 27116-27790 | 675 |
| E | 27864-28112 | 249 |
| M | 28127-28786 | 660 |
| N gene | 28842-30128 | 1287 |
| ORF8b | 28887-29493 | 609 |
| 3'UTR | 30133-30481 | 348 |
